# Enhanced maximal exercise capacity, vasodilation to electrical muscle contraction, and hind limb vascular density in ASIC1a null mice

**DOI:** 10.14814/phy2.13368

**Published:** 2017-08-07

**Authors:** Heather A. Drummond, Lusha Xiang, Alejandro R. Chade, Robert Hester

**Affiliations:** ^1^ Department of Physiology and Biophysics and the Center for Excellence in Cardiovascular‐Renal Research University of Mississippi Medical Center Jackson Mississippi

**Keywords:** Degenerin, functional hyperemia, ion channel, maximal oxygen consumption

## Abstract

Acid‐sensing ion channel (ASIC) proteins form extracellular proton‐gated, cation‐selective channels in neurons and vascular smooth muscle cells and are proposed to act as extracellular proton sensors. However, their importance to vascular responses under conditions associated with extracellular acidosis, such as strenuous exercise, is unclear. Therefore, the purpose of this study was to determine if one ASIC protein, ASIC1a, contributes to extracellular proton‐gated vascular responses and exercise tolerance. To determine if ASIC1a contributes to exercise tolerance, we determined peak oxygen (O_2_) uptake in conscious ASIC1a^−/−^ mice during exhaustive treadmill running. Loss of ASIC1a was associated with a greater peak running speed (60 ± 2 vs. 53 ± 3 m·min^−1^, *P *= 0.049) and peak oxygen (O_2_) uptake during exhaustive treadmill running (9563 ± 120 vs. 8836 ± 276 mL·kg^−1^·h^−1^, *n *= 6–7, *P *= 0.0082). There were no differences in absolute or relative lean body mass, as determined by EchoMRI. To determine if ASIC1a contributes to vascular responses during muscle contraction, we measured femoral vascular conductance (FVC) during a stepwise electrical stimulation (0.5–5.0 Hz at 3 V for 60 sec) of the left major hind limb muscles. FVC increased to a greater extent in ASIC1a^−/−^ versus ASIC1a^+/+^ mice (0.44 ± 0.03 vs. 0.30 ± 0.04 mL·min^−1^·100 g hind limb mass^−1^ · mmHg^−1^, *n *= 5 each, *P *= 0.0009). Vasodilation following local application of external protons in the spinotrapezius muscle increased the duration, but not the magnitude, of the vasodilatory response in ASIC1a^−/−^ mice. Finally, we examined hind limb vascular density using micro‐CT and found increased density of 0–80 *μ*m vessels (*P *<* *0.05). Our findings suggest an increased vascular density and an enhanced vasodilatory response to local protons, to a lesser degree, may contribute to the enhanced vascular conductance and increased peak exercise capacity in ASIC1a^−/−^ mice.

## Introduction

Acid‐sensing ion channel (ASIC) proteins are neuronal members of the degenerin protein family. As the name implies, ASIC channels are gated by decreases in extracellular pH and thus considered potential extracellular proton sensors. Most ASIC proteins form homo‐ and heteromeric channels that predominantly conduct inward Na^+^, but also conduct other cations. At least six ASIC proteins, encoded by four genes, have been identified: ASIC1a, ASIC1b, ASIC2, ASIC3, ASIC4, and BLiNaC (Duan et al. 2011; Price et al. 1996; Waldmann et al. 1996; Garcia‐Anoveros et al. 1997; Lingueglia et al. 1997; Chen et al. 1998; Ishibashi and Marumo 1998; de Weille et al. 1998; Babinski et al. 1999; Akopian et al. 2000; Grunder et al. 2000; Bassler et al. 2001; Wang et al. 2006). The crystal structure of ASIC1a suggests ASIC proteins form trimeric channels (Zeller and Skalak 1998). The pH sensitivity of ASIC channels varies considerably with the pH activation threshold ranging from pH_0.5_ of 4.3–6.8 (Kellenberger and Schild 2002; Wemmie et al. 2006). ASIC1a is one ASIC protein that may play a physiologic role in extracellular proton‐mediated responses because ASIC1a containing channels are sensitive to extracellular protons near the physiologic range (pH_0.5_ **= 6.1–6.8) (Benson et al. 2002; Hattori et al. 2009).

Several recent studies suggest that ASIC1a containing proteins and channels are also expressed in vascular smooth muscle cells (VSMC) (Grifoni et al. 2008a, 2008b; Jernigan et al. 2009; Chung et al. 2011). However, the physiological importance of VSMC protongated ASIC1a containing channels in vascular function is still unclear. Since ASIC1a containing channels are gated by extracellular protons, it is possible that VSMC ASIC1a containing channels may be involved in responses that are mediated by increases in extracellular protons, such as intense exercise.

Local extracellular acidosis (lactic acid and PCO_2_) associated with strenuous skeletal muscle contraction contributes to exercise hyperemia by a direct inhibitory effect on vascular tone that promotes local vasodilation and activation of Type III/IV afferent nerve endings that mediate increases sympathetic activation increased blood pressure, a response known as the metaboreflex (Sarelius and Pohl 2010). Interstitial pH in intensely contracting skeletal muscle has been reported as low as 6.4 (Victor et al. 1988; Street et al. 2001; Juel 2007), near the pH_0.5_ of ASIC1a. Thus, it is possible that ASIC1a containing channels may participate in the hyperemic response that occurs with intense exercise by mediating direct effects on vascular tone or muscle afferent activation. In the current study, we focused on the role of ASIC1a in vascular function because evidence suggests a role for ASIC3 in mediating muscle afferent activation; ASIC3‐like currents are present in more than 90% on neurons innervating the gastrocnemius, whereas ASIC1‐like currents are present in less than 10% (Xing et al. 2015). Since decreases in extracellular pH gate ASIC1a containing channels that conduct cations (Na^+^>Ca^2+^≫K^+^) inward, activation of such a channel might be expected to favor depolarization and vasoconstriction, rather than vasodilation. Therefore, we considered the possibility that ASIC1a channels in the vasculature might be counter‐regulatory and act as a brake on skeletal muscle vascular dilatory responses and potentially limit exercise capacity.

Therefore, the purpose of this study was to test the hypothesis that ASIC1a contributes to peak exercise capacity and regulates skeletal muscle vascular responses to muscle contraction and extracellular protons. To test this hypothesis, we measured (1) peak O_2_ uptake, CO_2_ output and running time/speed during exhaustive treadmill running, (2) hind limb conductance during electrical stimulation of the hind limb to mimic strenuous exercise, (3) local vasodilatory responses to external protons, and (4) hind limb vascular density in the ASIC1a null mouse model.

## Methods

All protocols and procedures described were reviewed and approved by the Institutional Animal Care and Use Committee of the University of Mississippi Medical Center.

### Knockout mouse model

Studies were conducted in genetically modified mice. Heterozygote ASIC1a^+/−^ mating pairs were generously provided by Michael J. Welsh, MD, at the University of Iowa. Genotypic analysis of offspring from heterozygous mating pairs was screened by polymerase chain reaction (PCR). Tail DNA was isolated using direct PCR (Tail, Viagen Biotech, Los Angeles, CA) at 3 weeks of age. PCR reactions were performed with AccuPrime Supermix (Invitrogen, Carlsbad, CA). Oligonucleotide sequences for the wild‐type allele were 5′‐TCTCCTATGAGCGGCTGTCT‐3′ and 5′‐GTCCGTCCCATTCCCTAAGT‐3′. Oligonucleotide sequences for the knockout allele were 5′‐GCCAGAGGCCACTTGTGTAG‐3′ and 5′‐GTCCGTCCCATTCCCTAAGT‐3′. DNA specific to wild‐type and knockout alleles was amplified using a Stratagene Robocycler under the following conditions. Samples were held at 94°C for 2 min, then cycled at 94°C for 30 sec, 55°C for 30 sec, 68°C for 40 sec, for 35 cycles, then held at 68°C for 5 min. PCR products were separated on agarose gels and visualized with GelRed Nucleic Acid Stain (Biotium, Hayward, CA). Wild‐type littermates of ASIC1a knockout mice were used as controls in all experiments. Animals were provided standard rodent chow and water ad libitum. Genotypes were reconfirmed following phenotypic analysis using liver samples. Animals were exposed to 12‐h light (06:00–18:00 h)–dark (18:00–06:00 h) cycles.

### Protocol 1: Detection of ASIC1a protein in VSMCs from femoral artery and its branches

To confirm ASIC1a expression in vascular tissue, we used amplified immunofluorescence in enzymatically dissociated femoral artery VSMCs. VSMCs were enzymatically dissociated in a manner previously described for renal and cerebral VSMCs and immunostained using our previously described approach for tyramide amplification (Molecular Probes, Eugene, OR) (Jernigan and Drummond 2006; Gannon et al. 2008; VanLandingham et al. 2009). Briefly, VSMCs were plated on glass slides, allowed to adhere, then fixed in 4% paraformaldehyde. Following a physiological buffer solution (PBS) rinse, the samples were treated with 3% H_2_O_2_ for 30 min, blocked in 1% tyramide blocking solution (TBS) for 1 h, then incubated with rabbit anti‐ASIC1a (1:100, Millipore) or goat anti‐ASIC1 (1:100, E‐15, Santa Cruz) and mouse anti‐*α* smooth muscle actin (1:100, Sigma) in 1% TBS overnight at 4°C in a humidified chamber. Samples were rinsed with PBS and incubated with Cy3‐conjugated donkey anti‐mouse IgG (1:200, Jackson Immunologicals) and donkey anti‐rabbit or anti‐goat HRP (1:200, Jackson Immunologicals) in 1% TBS for 1 h at room temperature protected from light. Samples were then rinsed with PBS and treated with Alexa 488‐conjugated tyramide according to the manufacturer's instructions for 10 min. Samples were then rinsed, mounted with coverslips, and imaged using a Leica TCS‐SP2 laser scanning confocal microscope. All samples were imaged side by side under identical collection conditions. Images were prepared identically in Photoshop.

### Protocol 2: Exercise capacity

Peak O_2_ uptake, CO_2_ output, and run time/speed were used as indexes of exercise capacity. For these experiments, mice were run on a treadmill (Columbus Instruments, Columbus, OH) to near exhaustion. Mice (10.8 ± 0.7, 9.4 ± 0.9 weeks of age for ASIC1a^+/+^ and ^−/−^ mice, *P *= 0.24, respectively) were allowed to acclimatize to the treadmill environment for 5 min. Following a 12‐min baseline collection period, the treadmill speed (0° incline) began at 6 m/min and was increased incrementally 2 m/min every 30 sec until near exhaustion. Mice were encouraged to run by tapping the Plexiglass enclosure and providing a mild shock from an electric grid at the end of the treadmill lane. The treadmill test was stopped when the animal would tolerate an electric shock for 5 sec.

### Protocol 3: Determination of body composition

We used magnetic resonance imaging (EchoMRI, Houston, TX) to measure lean and fat body mass to determine if increased exercise capacity in ASIC1a^−/−^ mice was due to a greater absolute or relative lean muscle mass. EchoMRI has been used previously to measure whole body composition in rodents (Nixon et al. 2010; Galgani et al. 2011).

### Protocol 4: Functional hyperemia to electrical hind limb stimulation in ASIC1a mice

For the measurement of femoral blood flow (FBF) responses to electrical stimulation, mice were maintained under isoflurane anesthesia on a heating pad to maintain body temperature at 37°C (rectal) for the duration of the study. The depth of anesthesia was monitored by the response to toe pinching. Mice were instrumented with a fluid‐filled carotid catheter for blood pressure measurement. The left femoral artery was carefully dissected free from the femoral vein at the exit from the abdominal wall to the epigastric artery. A perivascular flow probe (0.5 PSB, Transonic, Ithaca, NY) was carefully positioned to measure FBF. The left ankle was immobilized and stimulating electrodes were placed in the anterior and posterior portions of the thigh. A single test pulse of 3 V at 3 Hz was applied to check for muscle contraction. After a 10‐min stabilization period, a graded electrical stimulation protocol was initiated to mimic strenuous exercise. The electrical stimulation protocol consisted of a 3 V, 10 msec stimulus delivered at 0.5, 1.0, 2.0, 3.0, and 5.0 Hz for 1 min each, followed by 10 min recovery (Fig. 3A). FBF was recorded continuously using an ultrasound transit time flow meter (TS420, Transonic, low‐pass filter 30–40 Hz), along with blood pressure, with a computerized chart recorder (LabChart 6.0, PowerLab, ADInstruments, Colorado Springs, CO). Blood pressure was reported as mean arterial pressure (MAP). MAP and FBF data were recorded at 1000 Hz. FBF was normalized to mass of the hind limb and reported as mL·min^−1^·100 g hind limb weight^−1^. FBF was obtained in 5 −/− (1 female, 4 males) and 5 +/+ (5 males) littermate controls.

### Data analysis for FBF regulation with electrical stimulation

Femoral vascular conductance was calculated as FBF/MAP and reported as femoral vascular conductance (FVC, mL·min^−1^·100 g hind limb mass^−1^·mmHg^−1^). To quantify the effect of stimulation on MAP, FBF, and FVC, the last 10 sec of each period (baseline, stimulation steps, recovery) were extracted and averaged in LabChart and exported to Microsoft Excel for further analysis.

### Protocol 5: Vasodilation to local protons (H^+^ ions) in ASIC1a mice

For these studies, mice were anesthetized and the spinotrapezius muscle and surface vasculature visualized. The muscle was superfused with warmed external solution (37°C) of the following composition (millimolar concentrations): 118.07 NaCl, 6.17 KCl, 2.55 CaCl_2_, and 25 NaHCO_3_. The solution temperature is maintained at 35°C, equilibrated with gases containing 5% CO_2_, 95% N_2_, and the pH adjusted to 7.35–7.45. For the pH 6.5 solution, the control solution was adjusted using 2 N HCl (Xiang et al. 2005). Osmolarity of the pH 7.4 and 6.5 solutions were measured at 292 and 297 mOsm, respectively, using a *μ*Osmette (Precision Systems). Solution pHs were measured at start to end of protocol. Arcade vessels were identified and control responses to adenosine (100 *μ*mol/L) were determined. Vascular responses to extracellular protons (external solution at pH 6.5) were normalized to maximal dilation with adenosine. Internal diameter measurements were obtained before and during exposure to extracellular protons at 1 min intervals for 5 min.

### Protocol 6: Micro‐CT quantification of hind limb vascular density in ASIC1 mice

For these experiments, mice were anesthetized with isoflurane for placement of a retrograde carotid artery catheter. The superior mesenteric, celiac, and renal arteries were ligated to direct the vascular dye toward the lower limbs. The liver was snipped to allow escape of excess circulatory volume due to subsequent saline plus heparin flush and infusion of 2 mL Microfil dye (MV122, FlowTech) diluted (1:1:0.1, Microfil, diluent, curing agent; 10 mL total), infused at a rate of 1 mL/min using an infusion pump. Following the infusion, the catheter was tied and the mouse placed +4°C for 24 h to allow the Microfil to set. The specimen was placed in 4% paraformaldehyde for at least 72 h before scanning. Micro‐CT scanning was performed using a Skyscan 1076 bench micro‐CT (Bruker). The samples were positioned in a rotatable stage and scanned at 0.3° angular increments at a resolution of 9 *μ*m. Image display and analysis of vascular density was performed using the ANALYZE™ (Mayo Clinic, Rochester, MN) software package. (Iliescu and Chade 2010; Iliescu et al. 2010).

Mice were used between 8 and 26 weeks of age. All wild‐type and null mice were age matched for each protocol and there was no difference in age or body weight between genotypes within any protocol. Following every terminal procedure, a sample of the liver is collected to obtain DNA to confirm the animal genotype by PCR.

### Statistics

All data are presented as mean ± standard error. Differences between groups were identified using a one‐ or two‐tailed *t*‐*t*est or using a two‐way repeated measures analysis of variance followed by the Student–Newman–Keuls post hoc test, where appropriate. Significance was set at *P *<* *0.05. Specific *P* values are provided for certain datasets to demonstrate the high level of confidence in our statistical analyses.

## Results

### ASIC1a expression in vascular tissue of skeletal muscle

**Figure 1:**
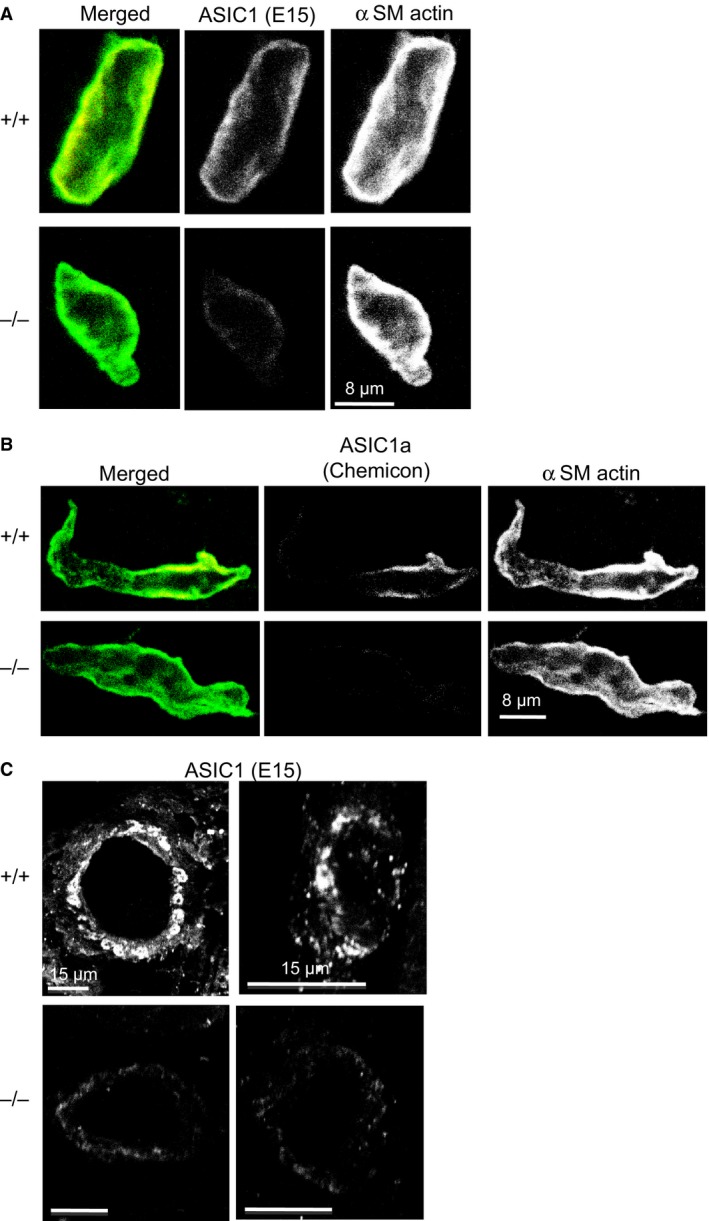
ASIC1 protein is expressed in hind limb VSMCs. VSMCs were labeled with SM *α*‐actin (left panel) and two different ASIC1 antibodies (middle panels). The merged images are shown in right panel. A low level of background staining was observed in the ASIC1a^−/−^ VSMCs, however, significantly more labeling was observed in ASIC1a^+/+^ VSMCs using both antibodies.

To determine expression of ASIC1a, we used immunolabeling in dissociated femoral artery VSMCs isolated from an ASIC1a^+/+^ and ASIC1a^−/−^ animal to determine if ASIC1a protein is expressed in femoral VSMCs. As shown in Figure 1, we detected expression of ASIC1 in *α*‐SM actin‐labeled VSMCs (left panels) using two different commercially available ASIC1 antibodies (middle panels). The merged images are shown in the left panel, and yellow coloration indicates colocalization of ASIC1 and *α*‐SM actin. Figure 1A represents staining with an ASIC1 antibody directed to the C‐terminus (E15). This antibody does not distinguish between the ASIC1a and 1b variants. Panel B represents staining with an antibody directed to the N‐terminus (Chemicon) and specifically recognizes the ASIC1a variant. The ASIC1 signal is present at or near the VSMC membrane in ASIC1a^+/+^ VSMCs. These findings suggest ASIC1a is present in VSMCs. ASIC1 labeling was also detected in VSMCs of small arteries (Fig. 1C left, ~30 *μ*m diameter) and arterioles (right, <15 *μ*m diameter) from frozen tissue sections. The small signal remaining in the ASIC1a^−/−^ VSMCs and vessels shown in Figure 1A and C likely represents ASIC1b since the C‐terminal antibody (E15) also detects the ASIC1b variant, which is not targeted in the ASIC1a^−/−^ model (Wemmie et al. 2002).

### ASIC1a opposes peak O_2_ uptake, CO_2_ output, and running time/speed during exhaustive treadmill running

**Figure 2:**
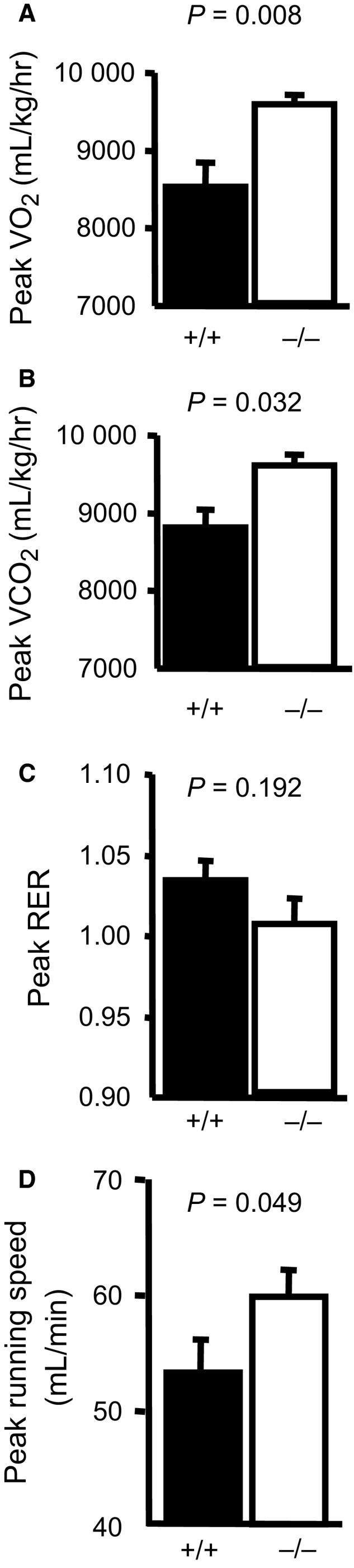
ASIC1a^−/−^ null mice have a greater exercise capacity and peak VO_2_ uptake. Using a standard treadmill running test, ASIC1a^−/−^ mice (26 ± 2 g body weight, 11 ± 1 week of age) have a greater peak oxygen uptake (A, E) and peak CO_2_ output (B, F) than ASIC1a^+/+^ littermates (25 ± 1 g, 10 ± 1 week). The RER response during treadmill running was similar (C, G). ASIC1a^−/−^ mice had a greater peak running speed (D, H). *n *= 6–7 each group. Actual *P* values are displayed to denote confidence in statistical analysis.

The age, body weight, resting O_2_ uptake, CO_2_ output, and respiratory exchange ratio (RER) were not different between ASIC1a^+/+^ and ASIC1a^−/−^ mice (Table 1). The peak O_2_ uptake (Fig. 2A), CO_2_ output (Fig. 2B), RER (Fig. 2C), and peak running speed (Fig. 2D) during exhaustive treadmill running in ASIC1a^+/+^ and ASIC1a^−/−^ mice are shown. Peak O_2_ uptake and CO_2_ output indicate increased exercise capacity in ASIC1a^−/−^ mice. The similar RER suggests mice had a similar utilization of glucose and lipids.

**Table 1:** Characteristics of ASIC1a mice used in maximal oxygen consumption studies

	ASIC1a genotype		*P* value (two‐tailed)
	+/+	−/−	
Resting VO_2_ (mL·kg^−1^·h^−1^)	5888 ± 252	6154 ± 268	0.32
Resting VCO_2_ (mL·kg^−1^·h^−1^)	4854 ± 188	5134 ± 306	0.36
RER	0.826 ± 0.009	0.832 ± 0.023	0.95
*n*	7	6	
M:F	6:1	5:1	

RER, respiratory exchange ratio.

### ASIC1a does not influence absolute or relative lean muscle mass

Since O_2_ utilization and work capacity are related to muscle mass, we wanted to determine if a larger muscle mass might contribute to the higher O_2_ uptake in ASIC1a^−/−^ mice. We used ECHO MRI to assess absolute and relative fat and lean body mass in the mice. As shown in Table 2, there were no differences in absolute or relative lean or fat mass in ASIC1a^−/−^ (*n* = 6) and ASIC1a^+/+^ (*n* = 11) mice. This finding suggests the increased O_2_ utilization and work capacity in ASIC1a^−/−^ mice is not due to increases in absolute or relative lean muscle mass.

**Table 2:** ECHO MRI assessment of body composition of ASIC1a mice

	ASIC1a genotype		*P* value (two‐tailed)
	+/+	−/−	
Body weight (g)	29.8 ± 0.9	29.4 ± 0.2	0.769
Age (weeks)	15.4 ± 0.5	15.6 ± 0.5	0.797
Lean body mass (LBM, g)	24.4 ± 0.6	23.7 ± 0.2	0.474
LBM (% BM)	82.3 ± 1.6	80.9 ± 0.4	0.635
Fat mass (FM, g)	3.3 ± 0.6	3.6 ± 0.4	0.743
FM (% BM)	10.8 ± 1.7	12.1 ± 0.5	0.654
Free H_2_O (g)	0.16 ± 0.02	0.13 ± 0.12	0.229
*n*	11	6	
M:F	11:0	6:0	

### ASIC1a opposes increases in femoral blood flow and conductance during strenuous muscle contraction

**Figure 3:**
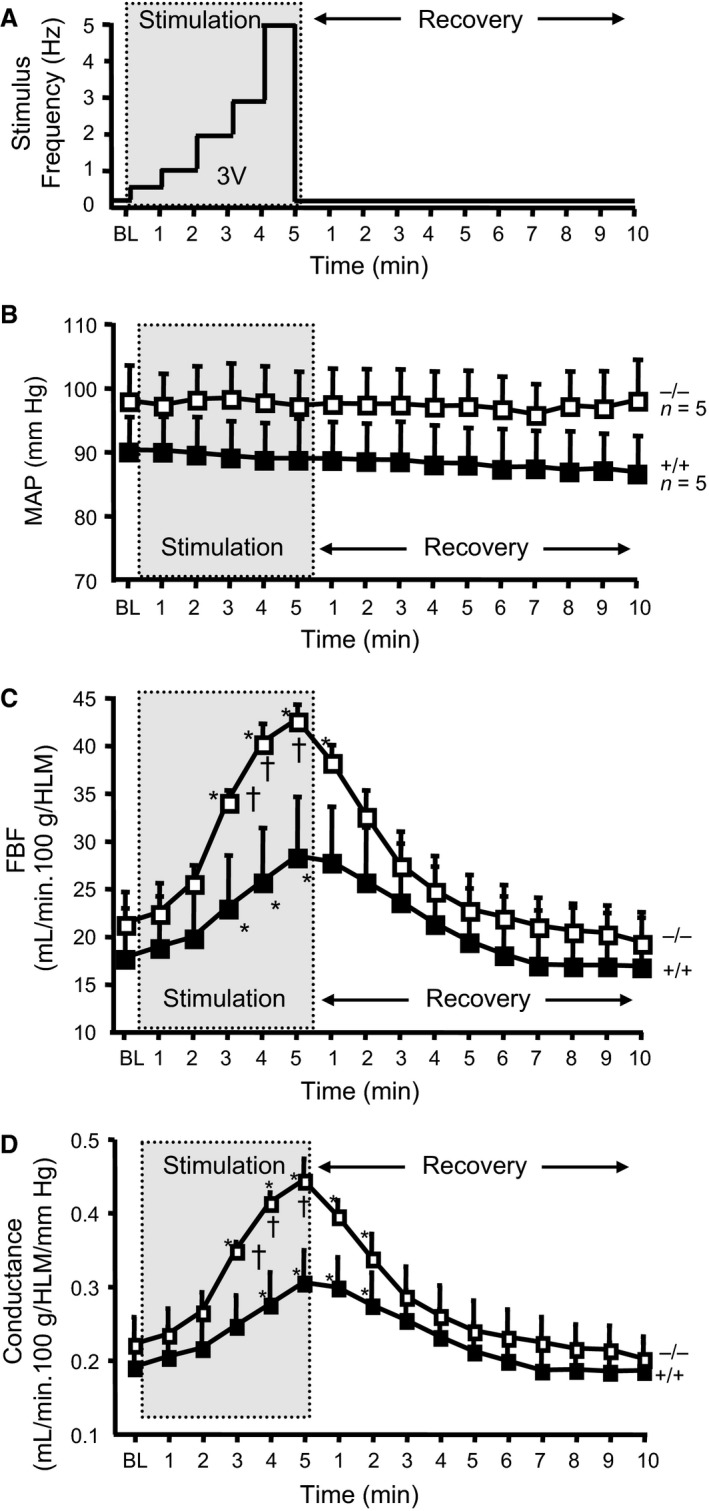
ASIC1a^−/−^ null mice have an enhanced hind limb blood flow response to simulated intense short‐term exercise. (A) The electrical stimulation protocol used to simulate exercise is shown. Hind limb muscles were activated with a 3‐V stimulus delivered at 0.5, 1, 2, 3, and 5 Hz for 1 min each step. MAP (B), FBF (C), and femoral conductance (D) responses for baseline (BL, 1 min), stimulation (5 min), and recovery (10 min) are shown. ASIC1a^−/−^ (*n *= 5, 36 ± 2 g body weight, 23 ± 2 weeks of age had a greater peak hind limb blood flow and vascular conductance during the last three steps of stimulation compared to ASIC1a^+/+^ mice (*n *= 5, 35 ± 2 g, 18 ± 2 weeks). *Significantly different from BL within genotype, *P *<* *0.05. ^†^Significantly different from +/+ at respective time point, *P *<* *0.05. Two‐way repeated measures ANOVA for conductance: genotype (g)* *= 0.260, time point (tp) <0.001, g × t *P *<* *0.001.

To determine if ASIC1a contributes to the hyperemic response in skeletal muscle with exercise, we used electrical stimulation of the major hind limb muscles to mimic exercise and measured femoral artery blood flow in anesthetized mice. The protocol used to stimulate muscle contraction is shown in Figure 3A. The characteristics and resting hemodynamic parameters (MAP, FBF, FVC) are shown in Table 3. There was no difference in age, weight, or resting MAP, FBF, or FVC between ASIC1a^+/+^ (*n *= 5) and ASIC1a^−/−^ (*n *= 5) mice. As shown in Figure 3B, MAP was similar between ASIC1a^+/+^ and ASIC1a^−/−^ mice and did not change with exercise. During exercise, FBF (Fig. 3C) and FVC (Fig. 3D) progressively increased in both groups as contraction intensity increased and returned to control levels within 5 min following removal of electrical stimuli. Peak FBF (43 ± 2 vs. 28 ± 5 mL·min^−1^·100 g HLM^−1^, *P *= 0.013) and peak FVC (0.44 ± 0.03 and 0.30 ± 0.04 mL·min^−1^·100 g HLM^−1^·mmHg^−1^, *P *= 0.009) were significantly greater in ASIC1a^−/−^ mice compared to ASIC1a^+/+^ mice. Although one female animal was included in the ASIC1a^−/−^ group, its response was similar to the males. This finding suggests ASIC1a opposes increases in femoral blood flow during exercise.

**Table 3:** Baseline hemodynamic parameters in ASIC1a mice functional hyperemia experiments

	ASIC1a genotype		*P* value (two‐tailed)
	+/+	−/−	
HLM (g)	1.9 ± 0.1	1.7 ± 0.1	0.176
HLM/BW	4.9 ± 0.4	4.4 ± 0.2	0.256
MAP (mmHg)	93 ± 5	98 ± 6	0.539
FBF (mL·min^−1^·100 g HLM^−1^)	18 ± 4	21 ± 3	0.475
FVC (FBF/MAP)	0.19 ± 0.04	0.22 ± 0.04	0.571
*n*	5	5	
M:F	5:0	4:1	

HLM, hind limb mass; MAP, mean arterial pressure; FBF, femoral blood flow; FVC, femoral vascular conductance.

### Vasodilation to local externally applied protons is prolonged in ASIC1a^−/−^ mice

**Figure 4:**
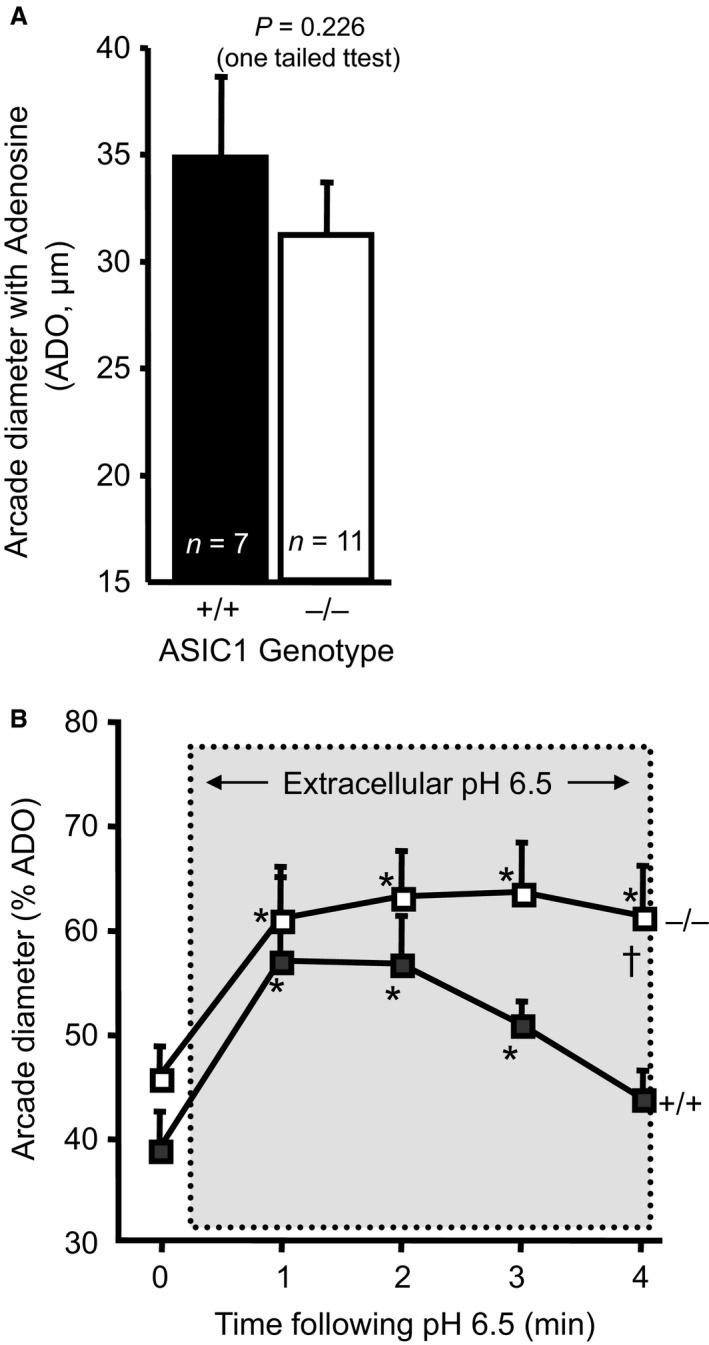
The vasodilatory response of arcade arteries to local application of protons in the spinotrapezius muscle is prolonged in ASIC1a^−/−^ mice. (A) Maximal vasodilation to adenosine (ADO, 100 *μ*mol/L) was similar between ASIC1a^+/+^ (*n *= 7 [5F, 2M], 25 ± 2 g body weight, 21.9 ± 2.8 weeks of age) and ASIC1a^−/−^ mice (*n *= 11 [7F, 4M], 27 ± 2 g, 19.4 ± 1.9 weeks). (B) Vasodilation to external application of pH 6.5 solution leads to transient increase in diameter in ASIC1a^+/+^ mice. The duration, but not magnitude, of the dilation to extracellular protons was increased in ASIC1a^−/−^ mice. *Significantly different from Time 0 (control) within genotype, *P *<* *0.05. ^†^Significantly different from +/+ at respective time point, *P *<* *0.05.

Since ASIC channels are gated by decreases in extracellular pH, we wanted to determine if the vasodilatory response to local protons in skeletal muscle might be augmented in ASIC1a^−/−^ mice and contribute to the enhanced hind limb blood flow response. To test this hypothesis, we determined local vasodilation of arcade arterioles to external protons in the spinotrapezius muscle in vivo. This muscle was used because it allows in vivo imaging of vascular diameter with the application of local external solutions. Resting diameters (13 ± 2 vs. 14 ± 1 *μ*m) and maximal vasodilatory responses to local adenosine (100 *μ*mol/L, 35 ± 2 vs. 32 ± 2 *μ*m) were similar between ASIC1a^+/+^ (*n *= 7; 5 M, 2F) and ASIC1a^−/−^ (*n *= 11; 4 M, 7F) groups (Fig. 4A). In response to local externally applied protons, the initial vasodilation to pH 6.5 was similar (19 ± 3 vs. 19 ± 1 *μ*m diameter; 57 ± 9 vs. 61 ± 5% of maximum vasodilatory response to adenosine). However, vessels from ASIC1a^−/−^ mice had a prolonged vasodilatory response to external pH 6.5 (Fig. 4B). In control mice, vascular diameter returned to control values within 4 min following exposure to low external pH, however, arcade vessels remain significantly dilated in ASIC1a^−/−^ mice (43 ± 3 vs. 62 ± 5% adenosine). These findings suggest that while arcade vessels from ASIC1a^−/−^ mice do not have an enhanced vasodilatory capacity to adenosine or pH 6.5, they do have a prolonged vasodilatory response to external pH 6.5.

### Hind limb vascular density is enhanced in ASIC1a^−/−^ mice

**Figure 5:**
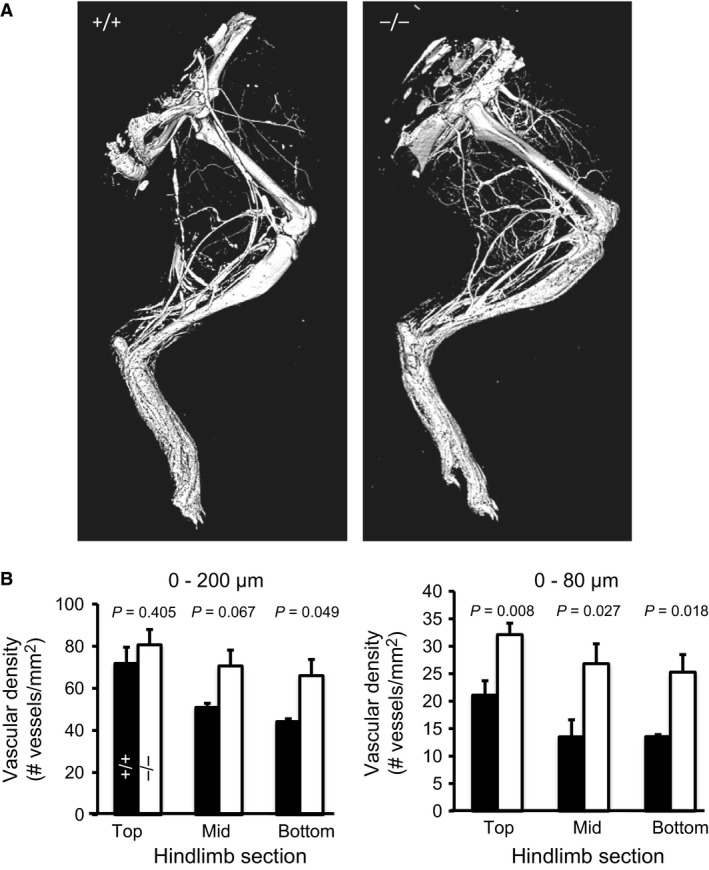
Increased vascular density in the hind limb of the ASIC1a^−/−^ mouse using micro‐CT. Representative images (A) and group data (B) of hind limb vasculature in ASIC1a^+/+^ (black columns, *n *= 3, 10.3 ± 0.1 weeks, 2M, 1F) and ASIC1a^−/−^ (white columns, *n *= 4, 10.9 ± 0.1, weeks, 2M:2F) mice. As shown in Panel B, the number of labeled small vessels under 80 *μ*m in diameter was increased in ASIC1a^−/−^ mice.

Since the difference in local vasodilatory response to external protons is not likely to entirely account for the differences in hind limb blood flow with electrical stimulation, we considered the possibility that structural differences in the hind limb vasculature might also contribute. To address this possibility, we determined hind limb vascular density using 3D micro‐CT reconstructions of the samples after microfil perfusion. Vascular density was visualized using micro‐CT. As shown in Fig 5, we found that density of small vessels (less than 80 *μ*m) in the hind limb was significantly increased in the ASIC1a^−/−^ mice.

## Discussion

ASIC proteins are members of a protein family, called degenerins, that are thought to act as chemo‐ and mechanosensors in certain neurons (Mano and Driscoll 1999; Bianchi and Driscoll 2002; Kellenberger and Schild 2002). Since ASIC channels are activated by drops in extracellular pH, they have been considered as potential chemosensors. Our laboratory has previously shown ASIC proteins are also expressed in VSMCs (Price et al. 2000; Drummond et al. 2004; Jernigan and Drummond 2005, 2006; Ge et al. 2012). A few recent studies demonstrate that proton‐gated currents attributed to ASIC1a channels are also expressed in VSMCs (Jernigan et al. 2009; Chung et al. 2010), yet, the physiological importance of these proton‐gated ASIC1a channels, as chemosensors or otherwise, in vascular function is not clear. We reasoned that ASIC1a containing channels might contribute to the hyperemic response associated with intense skeletal muscle contraction because (1) extracellular H^+^ ions have been postulated as a potential mediator of exercise‐induced hyperemia and (2) ASIC1a containing channels are gated in the interstitial pH range associated with intense exercise (Victor et al. 1988; Street et al. 2001; Juel 2007).

### Role of ASIC1a homomeric versus heteromeric channels

ASIC1a can form homomeric channels with a half‐maximal activation near the pH range (pH_0.5_ **= 6.6) (Hattori et al. 2009) with intense skeletal muscle contraction, ASIC1a can also form heteromeric channels with other ASIC proteins (ASIC2b, ASIC3) to form similarly pH‐sensitive channels (Benson et al. 2002; Hesselager et al. 2004; Hattori et al. 2009). Although we do not know the identity of ASIC channels in hind limb vasculature, we and others have shown that ASIC1a, 1b, 2a/b, and 3 are expressed in A10 cells, an aortic VSMC‐derived cell line, and cerebral and/or pulmonary VSMCs (Grifoni et al. 2008a; Jernigan et al. 2009; Chung et al. 2010). Thus, it is likely that small hind limb arteries and arterioles also express multiple ASIC proteins, making it difficult to distinguish the contribution of ASIC1a as homomeric versus heteromeric channel using the ASIC1a null model. Therefore, we can only conclude that ASIC1a containing channels contribute to the outcomes described here.

### Increased oxygen uptake in ASIC1a null mouse

To initially determine if ASIC1a contributes to vascular responses under conditions associated with extracellular acidosis, such as strenuous exercise, we first determined peak oxygen (O_2_) uptake in conscious ASIC1a^−/−^ mice during exhaustive treadmill running and found ASIC1a^−/−^ mice have a greater exercise capacity and a peak VO_2_ uptake than wild‐type littermates. We found that ASIC1a null mice tend to achieve a greater peak speed, O_2_ consumption, and CO_2_ production of approximately ~15%. A 15% increase in peak O_2_ consumption is comparable to the moderate–intense aerobic training or selection of mice based on high wheel running activity (Kemi et al. 2002; Rezende et al. 2006; Ericsson et al. 2010). In this study, we focused on the potential contribution of ASIC1a‐mediated vascular responses to the finding of increased peak O_2_ consumption in ASIC1a^−/−^ mice, however, other mechanisms, such as altered neural responses, may also contribute. However, they were not addressed here because they are beyond the scope of this study.

### How might ASIC1a contribute to exercise hyperemia response?

The hyperemic response associated with exercise is predominantly mediated by vasodilation and vascular recruitment following skeletal muscle contraction, which increases delivery of O_2_/nutrients and removal of CO_2_/metabolic waste. Several molecules have been suggested to mediate the vasodilatory response, including protons from the muscle metabolic by‐product lactic acid and pCO_2_. Thus, we hypothesized that ASIC1a may contribute to the vasodilatory response following intense skeletal muscle contraction by acting as a sensor of extracellular or interstitial protons. Most ASIC channels are permeable to Na^+^>K^+^>H^+^, however, ASIC1a channels are also permeable to Ca^2+^ suggesting that activation of ASIC1a might lead to membrane depolarization or activation of Ca^2+^‐dependent signaling (Waldmann et al. 1997; Yermolaieva et al. 2004; Hoagland et al. 2010; Sherwood et al. 2012). This led us to reason that ASIC1a may act as a brake on the hyperemic response in skeletal muscle, thus, ASIC1a^−/−^ mice might have a greater functional hyperemic response. As shown in Figure 3, the hyperemic response to a graded, intense hind limb muscle contraction was enhanced in the ASIC1a^−/−^ mouse, consistent with proton activation of ASIC1a channels blunting maximal vasodilation/recruitment.

### Potential underlying mechanism(s) for the enhanced hyperemic response

The mechanism(s) underlying enhanced flow responses typically include a greater vasodilatory capacity or recruitment/vascular density. However, our data do not support a greater vasodilatory capacity during exercise, that is, have a greater maximal dilatory ability or ability to dilate in response to external protons, in the ASIC1a^−/−^ model. Our data indicate the adenosine response and maximum dilatory response to pH 6.5 is similar between ASIC1a^+/+^ and ^−/−^ mice, which suggests that the maximum dilatory ability and response to extracellular protons of arcade arteries is similar. Due to the inclusion of several female mice in this protocol, a potential for gender bias exists and future studies are required to address this issue. Although we did find that dilation was prolonged in the ASIC1a^−/−^ mouse, it seems unlikely to account for the increased exercise capacity and hind limb flow.

### Does the increased osmolarity of the low‐pH solution contribute to the vasodilation in the spinotrapezius?

The addition of HCl to achieve the pH 6.5 solution resulted in a slightly higher osmolarity compared to the pH 7.4 solution (292 vs. 297 mOsm). Hyperosmolarity has been shown to induce vasodilation in skeletal and cardiac muscle arteries and arterioles (Ishizaka and Kuo 1997; Massett et al. 2000; de Clerck et al. 2005). However, the vasodilatory responses relative to the magnitude of the osmotic stimulus in these studies were much smaller than observed in the current investigation. For example, Massett et al. reported that a 5‐mOsm stimulus increased the diameter by roughly 5% in gracilis arterioles. de Clerck et al. reported a 60‐mOsm stimulus induced a 46% dilation in gluteal arterioles. In coronary arterioles, a 20‐mOsm stimulus increased diameter 30% (Ishizaka and Kuo 1997). In our study, the osmolarity of the pH 6.5 was only 5 mOsm greater than the pH 7.4 solution, yet elicited a 40–50% dilation in the spinotrapezius arcade arterioles, substantially greater than the dilation elicited by an equivalent hyperosmotic stimulus reported by Massett et al. Although we cannot rule out the possibility that the 5 mOsm increase of the 6.5 pH solution contributes, at least in part, to the vasodilation in Figure 4B, we can reason that it probably does not account for the entire response.

The reason for the prolonged response in the ASIC1a^−/−^ is unclear, however, it is possible that changes in ASIC channel desensitization properties may contribute. ASIC channels rapidly desensitize to prolonged extracellular proton exposure, which may explain the decline in vasodilation with continued exposure to extracellular protons in the wild‐type group (Mano and Driscoll 1999; Benson et al. 2002; Bianchi and Driscoll 2002; Kellenberger and Schild 2002; Hesselager et al. 2004; Gautam and Benson 2013). For example, the desensitization constant of ASIC2a channels is 1.4 sec, but decreases to 0.4 when coexpressed with ASIC1a (Hesselager et al. 2004). Thus, loss of ASIC1a may alter the channel desensitization properties (Benson et al. 2002; Hesselager et al. 2004; Hattori et al. 2009). Regardless of the mechanism underlying the prolonged vasodilatory response, the response itself does not seem sufficient to account for the significant increase in exercise capacity or hind limb blood flow in the ASIC1^−/−^.

Our data support enhanced recruitment, or vascular density, in the hind limb skeletal muscle as a potential mechanism underlying the enhanced exercise and hind limb blood flow response in the ASIC1a^−/−^ model. Using micro‐CT, we found an increased number of small vessels (<80 *μ*m) in the whole hind limb of ASIC1a^−/−^ mice. These findings have significant implications in the importance of ASIC1a, and possibly other degenerins, as important mediators of remodeling and adaptations of the microvascular networks.

### A role of ASIC1a in muscle afferents as H^+^ sensor?

The muscle metaboreflex is a neural reflex that contributes to increased muscle blood flow during exercise and/or ischemia. Thin afferent nerve endings in skeletal muscle are activated by increases in metabolites, including interstitial H^+^, which results in increases in phrenic and sympathetic nerve activity to increase ventilation and systemic blood pressure (Clifford 2011; Murphy et al. 2011; Oelberg et al. 1998; Smith et al. 2006; Xing et al. 2015). The increase in systemic blood pressure contributes to the increase in flow. Because ASIC channels are gated by extracellular H^+^ ions, they are potential mediators of the muscle metaboreflex. Evidence supports this: ASIC proteins are expressed in the sensory neurons that innervate skeletal muscle and pharmacological ASIC channel inhibition suppresses the exercise pressor reflex (Chen et al. 1998; Benson et al. 2002; Molliver et al. 2005; Hayes et al. 2007; McCord et al. 2009). However, it is unlikely that an ASIC‐mediated metaboreflex response contributes to the enhanced hyperemia observed in the ASIC1a^−/−^ model in the current investigation for several reasons. First, ASIC3, not ASIC1a, is thought to be the most likely metaboreflex mediator candidate. ASIC3 currents and protein levels are expressed in a greater population of skeletal muscle sensory neurons than ASIC1 (Molliver et al. 2005; Xing et al. 2015). ASIC3 like are expressed in 91% of dorsal root ganglion neurons innervating the gastrocnemius, while ASIC1a‐like currents are expressed in 9% (Xing et al. 2015). Second, blood pressure and heart rate (data not shown) does not change in either group during muscle stimulation. A metaboreflex‐mediated increase in sympathetic discharge would be expected to increase blood pressure and or heart rate. Third, the metaboreflex is activated under conditions of ischemia. Other than muscle contraction itself, there is no evidence of hind limb blood flow obstruction in our preparation as flow increases twofold in the control group. Based on these findings, it is unlikely that the metaboreflex is activated with electrical muscle contraction. However, based on the approaches used in the current study, we cannot rule out a role for ASIC1a in the muscle metaboreflex. Determining the contribution of the muscle metaboreflex to the hyperemic response with muscle contraction is beyond the scope of this investigation.

### Role of ASIC1 in neuromuscular fatigue?

Muscle fatigue is a complex process involving central and peripheral neural mechanisms, muscle metabolism, and local blood flow (Gandevia 2001; Allen et al. 2008; Ament and Verkerke 2009). A potential role for ASIC1a in muscle fatigue was suggested by a recent report from Urbano et al. (2014) showing ASIC1a^−/−^ female, but not male, mice had reduced forelimb strength (assessed by hang time when suspended from a wire) and enhanced neurotransmitter release from presynaptic motor neurons in isolated levator auris longus muscle (dorsal surface of the neck), suggesting ASIC1a may contribute to an inhibitory “fine tuning” of neurotransmitter release at the motor neuron terminal. In the current study, we observed a significantly greater exercise capacity with treadmill running in the ASIC1a^−/−^ mice. Although all but one subject in each genotype were males, the female followed the same pattern as the males: the ASIC1a^−/−^ female had a greater peak VO_2_, VCO_2_, and running speed compared to the wild‐type control. The mechanisms underlying the different responses to exercise in Urbano et al. and the current study (hang time vs. treadmill running) are unclear, however, they may reflect the importance of ASIC1a in muscle fatigue associated with gender (female vs. male), muscle contraction type (isometric vs. isotonic), muscle mass involvement (small vs. large), muscle fiber type (fast vs. slow) involvement, or the presence of an intact circulatory system. Thus, the role of ASIC1a in muscle strength and fatigue may be multifactorial (i.e., neurotransmitter release and local circulation to adjust O_2_ delivery/utilization) and further studies are needed to determine if muscle fatigue, or lack of, contributed to the responses in the current investigation.

### Significance

Taken together, findings of this study suggest ASIC1a plays an inhibitory role in vasodilation in response to external protons, functional hyperemia, and exercise capacity. Thus, ASIC1a does not mediate, but may oppose increases in vascular conductance with local protons and intense muscle contraction, perhaps through enhanced vascular recruitment and improved remodeling of the microvascular networks in skeletal muscle. Our finding that ASIC1a may play an important role in regulating vascular conductance with muscle contraction may prove to be a novel and interesting target in the treatment of vascular diseases associated with ischemia.

## Conflict of Interest

The authors have no conflicts of interest.
